# Pictographic Representations of the Word “Nature” in Preschool Education Children

**DOI:** 10.3389/fpsyg.2020.00575

**Published:** 2020-04-21

**Authors:** Blanca Silvia Fraijo-Sing, Norma Isabel Beltrán Sierra, César Tapia-Fonllem, Rosalba Valenzuela Peñúñuri

**Affiliations:** Department of Psychology, University of Sonora, Hermosillo, Mexico

**Keywords:** nature concept, kids drawings, preschool children, representations, physical environment

## Abstract

The relevance of preschool children’s understanding of nature, its elements, how it affects the behavior of human beings, and how human beings influence it, is a two-purpose task. First, it helps to identify the necessary elements for the design of programs that have a significant impact in the development of environmental identity. Second, it also assists in the implementation of environmental education in the school curriculum in Mexico, in order to develop attitudes to preserve the environment from an early age. Based on this logic, the objective of this study was to identify the components of the concept of nature and its relationship with environmental identity, from drawings made by preschool children in a desert environment through a visual discursive analysis. The sample consisted of 118 preschool students whose ages ranged between 5 and 6 years. Participants were selected from four different schools in Hermosillo, Mexico: three located in the urban area and one on the coastal area of the State of Sonora. Participants were asked to draw the first thing that came to their minds when they heard the word nature. As a result, all the drawings presented categories such as plants, animals, waterbodies, celestial bodies, abiotic factors, natural locations, locations made by man, and others. Finally, the analysis showed that a general idea of what nature represents to children includes elements of known flora and fauna; however, they did not capture elements of the desert region in which they live. In addition, most participants’ self-definition contained environmental identity.

## Introduction

The research of environmental psychology focuses on the study of the activity of the individual in their physical and social context in order to find logic on the connections between human beings and their environment. On one hand, it analyzes perceptions, attitudes, environmental assessments, and representations, and, on the other hand, environmental behavior (Moser, 2014). The environment also plays an important role in defining and expressing the identity of individuals, which is mediated through a complex pattern of beliefs, values, feelings, expectations, and preferences relevant to the physical world (Proshansky et al., 1983). An individual gives meaning to these environments according to the emotional impact that affects their cognitive, evaluative, and behavioral activity. These factors determine the level and the ways the subject is involved in each of these spaces.

This way, the physical space where an individual develops becomes a significant factor in the process of person-environment interaction, where an analysis of the psychological processes and environmental factors that participate in them is indispensable (Corraliza and Berenguer, 2000). Furthermore, attitudes toward the environment, such as concerns, also influence and reflect different values such as egoistic, altruistic, and biospheric attitudes. Given that the individual reflects their concerns on themselves, toward other people, or toward all living beings and ecosystems, these attitudes are reflected in the perception and behaviors toward the environment (Schultz, 2002).

Environmental studies in children are an adaptation of the studies carried out by adults; therefore, it is necessary to also consider the youngest population in order to know their first thoughts about environmental issues. These issues are highly related to environmental identity, for the way we perceive our environment, and especially how we act on it or from it, can provide insight into the phenomenon of understanding our identity and our immediate natural space. These concepts are constructed from spatial, sociocultural, temporal, and community bases on cognitive, evaluative, and behavioral processes (Zimmermann, 2010). These identity behaviors are formed from the first stages of development and within cultural, historical, and dynamic patterns, where relatively permanent meanings and representations are configured and reinforced during the following years (Delval, 2004; Rojas, 2004). Thus, it is important to explore environmental perceptions in children and obtain a starting point to, eventually, influence them through their school classes on issues about care and protection of the environment, as well as to generate attention, evaluation, and action in programs of environmental education (Clayton, 2012). As an example, Olivos et al. (2014) studied connectivity with nature, environmental identity, and pro-environmental behavior, where they found patterns of positive behaviors related to identity and connectivity with nature.

The study of the relationship of children with nature entails values at this stage of human life that relate to the characteristics of the natural environment (Myers, 2012). In children, development is intrinsically connected with the basic areas of psychology that include sensation and perception, spatial cognition, and, in some cases, nature-related psychopathologies such as phobias of animals (Clayton and Myers, 2015). According to the theory of human development formulated by Piaget (1964), it is in the preoperative stage where children begin to develop the ability to represent and perceive their physical environment, which makes them able to recognize their environment and act on it (Delval, 2004). The first years of life and the beginning of formal education is a relevant stage in the development of opportunities to act freely and learn to be responsible from an early age, which has been linked to prosocial behavior and cooperation. Furthermore, contact with natural environments is associated with well-being and connection with nature (Zhang et al., 2014; Zelenski et al., 2015; Sobko et al., 2018), which fosters the concern for the preservation of their environments. Moreover, environmental knowledge of preschool children can provide a new perspective that stimulates reflection on the individual’s relationship with nature and the active construction of new understandings (Selin, 2013). Finally, it is considered fundamental to use drawings to study preschool children’s representation of nature, because drawing helps children to express themselves about the real world that surrounds them (Wright, 2010).

## Children’s Drawings

Children can represent reality through different forms, and drawing is one of them, since it is an important cognitive component that aids children on reflecting about what they understand as reality, their spatial representation, and how they conceive things. Drawing is also considered a form of communication (Coates and Coates, 2011; Vivaldi and Salsa, 2017) and conveys an affective aspect (Delval, 2004). According to developmental theorists, said representations begin to take place from the age of two. This relates to the beginning of children’s primary socialization, either by their family environment or their school environment; therefore, drawings are a concrete and effective act that helps children record their perception from the world around them (Fox and Lee, 2013). This way, drawing has been used in various studies to collect information about perceptions and ideas in participants of different ages and in different themes. Additionally, drawing is also considered as a type of language in children, and, as Callaghan (2013) states, representations of children are a sign of the development process regarding the intention to communicate with other people.

Language, both oral and written, is a system of symbols. In oral language, the speaker must relate the oral symbol to a meaning or idea, both in reality and according to the perception of each individual. The same happens in written language, and, as children begin to write, they develop their relational principles because they must relate them to their own ideas, concepts, or meanings. Pictographic writing is children’s first approach to writing in an attempt to represent written language, objects, meanings, or conceptualizations (Brandt, 2015; Baroutsis et al., 2019).

Studies have shown that drawing is an appropriate strategy to gather information about how children perceive certain places, processes, or events. Some examples of studies utilizing this strategy are Baroutsis et al. (2019), where children were asked to draw how they explain the process of learning to write; Highet et al. (2019) measured the social impact of *H. pylori* in children through drawings, and also Moragón and Martínez (2016), who conducted a study to describe the way in which primary school children represent children’s play through drawings. The results concluded that drawings are a representation of the reality that children perceive, and the majority presented real elements about the research topic. However, it was also found that through drawing, children expressed their feelings and attitudes.

Moreover, other studies were found where it was intended to examine the perception of the natural environment, such as Günindi (2012) and Özsoy (2012), who conducted studies to examine the perception of the environment in preschool children through their drawings and the explanation that students gave about them. This was performed with the purpose of knowing how children build their thoughts and concepts. As a result, it was found that at least 81% of the children see a clean environment and 60% included people in their drawings, which shows that some children consider people as part of the environment. While the rest do not share this consideration of people, they do consider other living beings. Similar studies also evaluated how children perceived the environment in present and future times. In these cases, most of the students drew polluted technological environments using elements such as the sun, trees, humans, cars, and houses; some even showed robots and spaceships. Additionally, it was found that perception changed depending on the context where children were raised, and participants represented a greater number of favorable environmental elements other than the increase of temperature in the future and deteriorated forest and rivers (Pellier et al., 2014; Özsoy and Ahí, 2014).

On the other hand, Yilmaz and Kahraman (2015) and Yilmaz et al. (2012) analyzed the graphic productions of Turkish elementary students to determine how they reflect what they know about science and nature. The results showed that the words “science” and “nature” have some similar concepts as “environment.” However, science is explained with “laboratory environment,” while nature with “clean environment.” Another thing in common is that both words are related to living beings that are classified as “animals” and “plants,” but “human being” is excluded in both categories. Other than those already mentioned, the drawing technique was also used in a study related to the playground environment in a school (Salı et al., 2014), where children were asked to draw their dream playground and the one they had at the moment. It was found that children drew their ideal playground with movable and more interactive games. Macdonald (2009) mentions that the drawings and their previous oral description by the participants favor the holistic approach in research with children. Similarly, Angell et al. (2015) mention that graphic representations along with their oral description have been a central tool for researchers working with participants in the childhood stage due to the apparent simplicity, attractiveness, and disposition of the resources.

In this study, we focused on finding out how preschoolers represented what the word nature meant to them through drawing by emphasizing the objects and figures that children illustrate from their perception of nature, so that we could describe the relation with environmental identity. Considering that, in formal education in Mexico, there are few activities that present environmental education programs from the first school years, it is not considered as a variable that could affect student perception of environment. Conversely, other variables of the context such as the place of residence and family are.

## Method

### Sample

The sample size included 118 children aged between 5 and 6 at the time of the study, 59 females and 59 males, all enrolled in four different preschools. One of them was a private school, while the rest of them were public. The first school was located on the coast of the municipality of Hermosillo. From this school, 48 students participated, and the conditions of the classroom let the students work individually. The second school was a private school with a bilingual education system. From this school, 34 students participated, and the researchers worked in a hallway outside of the classroom to prevent participants from seeing their classmates’ work. The last two schools had the same dynamics; both were state-funded public schools with only one teacher for each class, located in an urban area. There were 16 participants from one school and 20 from the other. One was located at the south of the city, while the other one was up north. In them, work was done inside the classroom and only two tables and two chairs were available. The tables were placed as far away as possible from each other, and students were asked for their collaboration in the study. The students who accepted continued to carry out with their drawings, while their descriptions and annotations were written by the researcher.

The schools located in the urban area have limited vegetation, while the one located on the coast has very particular vegetation and fauna that predominate the landscape, such as saguaros, mesquites, trees, bushes, and a nearby beach. This region of Mexico is characterized by being desertic and having temperatures that exceed 45°C during the summer, while in winter temperatures can reach approximately 15°C or less.

### Survey and Data Analysis

Following the aforementioned logic, drawings were used as the main form of collecting information. Participants received white sheets, pencils, and colored crayons. They were subsequently asked, “What do you understand by nature?”, before being instructed to draw their answer on the sheet in front of them. The drawings were made individually so that there was no interference or influence by the responses of other classmates. Each student described their drawing while making it or after the fact, and no questions were made by the researcher or teachers. Then, the researcher wrote the descriptions down on a separate sheet of paper that was stapled to the drawing and then numbered (Nic Gabhainn and Kelleher, 2002).

Drawings, graphic representations, pictographs, or permanent products are a means to access the ideas, feelings, and experiences of children where the objects form part of a message regarding their understanding of the world (Macdonald, 2009; Fisher et al., 2014; Bland, 2015). In order to obtain relevant data in an investigation carried out with drawings, it is necessary to identify the components that capture the participant’s understanding of the specific topic through a content analysis (Krippendorff, 2013; Linder et al., 2017; Flores et al., 2018). The use of drawings and their keywords as tools allow the participants to understand the activity that they are asked to carry out despite of possible limitations in the stroke. In this fashion, they can express their thoughts in relation to a topic (Macdonald, 2009; Angell et al., 2015; Linder et al., 2017).

Along these lines, and after analyzing each drawing, the elements were codified, categorized, and described according to their components, which resulted in six different categories that appeared frequently in each of the drawings. It is worth noting that there were also drawings that did not fit into any category. The categorization presents an intra-coding reliability since the coding was carried out at first when classifying the elements according to their values and a second time after reviewing the literature to compare them with previous studies. In the same way, an intercoding was performed, since the categories were verified by experts in the subject and relevant changes were suggested for better results. For the content analysis, the words were classified into different thematic categories using the bottom-up strategy, so the categories were not previously established. The definition of common thematic categories is useful for comparisons between different case studies because it provides a systematic way of classifying perceptions (Maneja-Zaragoza et al., 2013).

Likewise, we used the visual discourse analysis of Albers (2007). This mentions that drawings are made up of support systems that indicate how they should be read considering the spatial elements and the distribution previously discussed by Zabulis and Orphanoudakis (2001) and Kress and Van Leeuwen (2007). The authors explain that each graphic composition has a visual attention center that does not necessarily correspond to the center of the paper, so they propose to divide it in quadrants to identify the elements with greater emphasis following the directionality provided by the text itself, which also does not correspond necessarily to the conventional directionality of writing. Thus, after obtaining the frequencies of the presented objects, the support systems were identified using both the children’s discourse and drawings, subsequently identifying the intention of the drawing and its central component. According to Albers (2007), the support systems are the ones available for reading the exchange of meanings between the creator and the interpreter based on the semantics of the text by means of the symbols used to represent the phenomenon within the cultural canons. Hence, visual representations are loaded with properties and cannot be reduced only to substitutions of the object in real life (Banks and Zeitlyn, 2015), so they present complementary properties that can be categorized into sets that reflect social processes and have a certain intention behind them.

## Results

The drawings generated by the participants showed symbols that referred to elements of nature; most of them were understandable and corresponded to the skills and knowledge expected in this stage of development. However, the rest of the works were rather abstract for the interpreters, so the oral descriptions made by the participants became an essential part in their categorization. In the collected drawings, there was a total of 133 elements obtained that derived in nine categories according to their similarity and mutually exclusive logic. Subsequently, another encoding was performed to enable a second analysis of the data obtained with this technique. Six different categories were found: locations, plants, animals, abiotic elements, bodies of water, and people (see Table 1).

**TABLE 1:** Coding scheme and description of the study categories.

Categories	Description	Category elements
Location	Place or environment in which the elements referred to the given task	Forest, beach, jungle, house, Hermosillo, zoo, island, city, land, jungle, hospital, school and garden.
Plants	Flora and vegetation specifically originated in the location.	Trees, flowers grass/grass, palm/palm, shrub, leaves, apples, pines, pineapple, bushes, algae, bananas, coconuts, seeds, corn and pumpkin.
Animals	Wildlife identified in the location	Butterfly, bird, dog, lion, fish, rabbit, snake, giraffe, monkeys, bears, elephant, tiger, duck, cat, wolf, bees, owl, squirrel, spiders, eagle, frog, gorilla, shark, jellyfish, bird, horse, jaguar, puma, leopard, kangaroo.
Abiotic Elements	Spatial arrangement between heaven and earth or other elements of the category.	Sun, sky, clouds, earth, rocks, world, mountains, sand, mud, air, moon, stars, rainbows, nest, rain and moth.
Water Bodies	Bodies of water in a similar spatial arrangement with elements related to the category.	Sea, river, lake, pond, water that sells from a tube, puddle and water to water plants.
People	Representations of themselves, identical people.	Himself, mom, boy/girl, dad, person, grandfather, cousins, grandmother, humans, hunters.

They found that the “animals” category had the highest frequency, since it was present 171 times in the 118 drawings. Particularly, the referred animals were butterflies, birds, dogs, lions, fish, rabbits, snakes, and giraffes; it is worth noting that some of the depicted animals are not typical of the participants’ own region. On the other hand, as can be seen in Table 2, bodies of water were only observed 29 times, with places such as seas, rivers, lakes, and ponds; however, only two participants from the coast area drew the sea. As for the location category, there were places that, like animals, are not typical in the region where the study was conducted, such as forests and jungles. Finally, the human factor was the second less frequent element presented (40 times), in which the participants referred to themselves, their parents, or other children.

**TABLE 2:** Number of drawings coded in each category.

Category	Frequency of referred elements	Most frequently referred category elements	Total of drawings
Location	41	Beach, forest, jungle and home.	118
Plants	134	Trees, flowers, grass and palms.	
Animals	171	Butterfly, bird, dog, lion, fish, rabbit, snake and giraffe.	
Abiotic elements	105	Sun, sky, clouds and earth.	
Water bodies	29	Sea, river, lake and pond.	
People	40	Me (the participant) mom, children and dad.	

**FIGURE 1:**
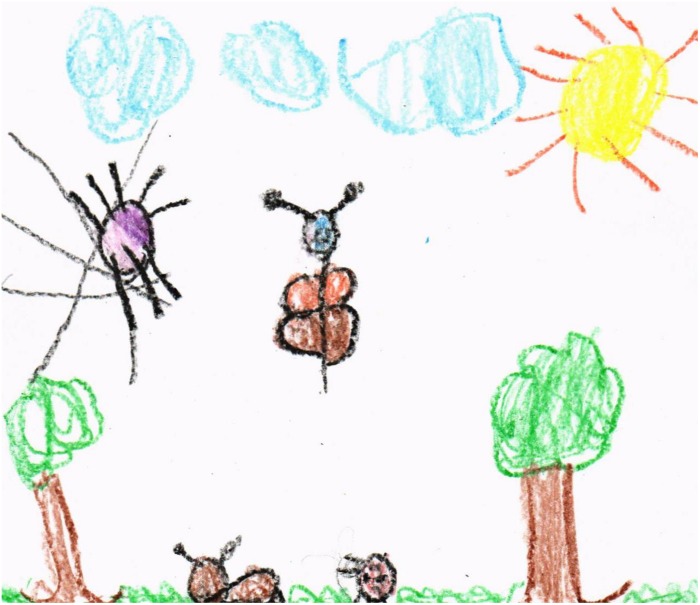
Categorized drawings.

**FIGURE 2:**
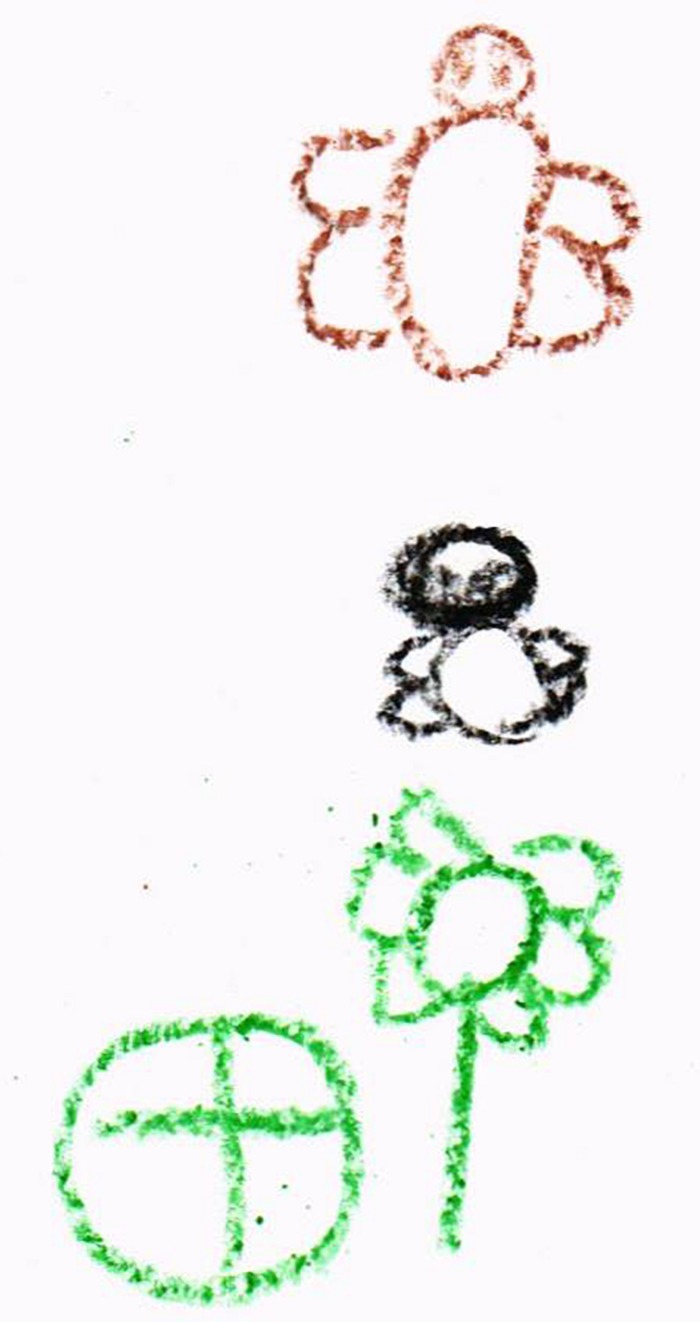
Drawing without proper symbolic representation.

**FIGURE 3:**
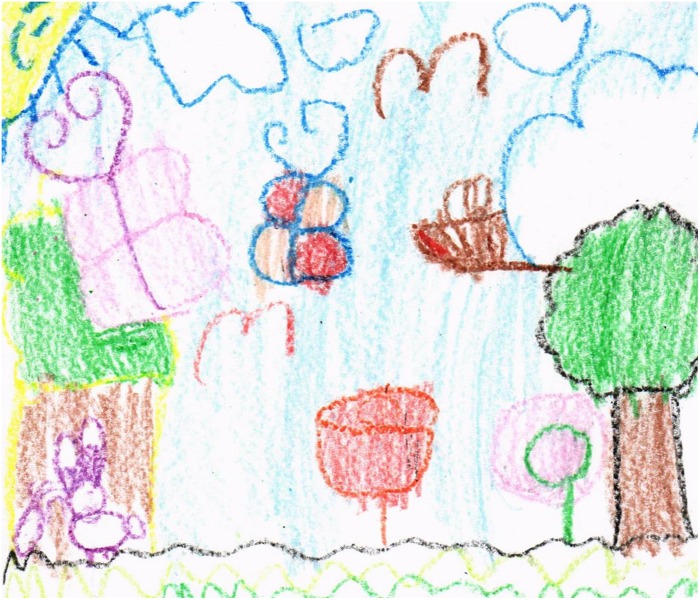
Drawing with proper symbolic representation.

As for the drawings themselves, there were some where it was possible to encode different categories, such as plants, animals, and abiotic elements, where each of them could be clearly spotted (see Figure 1). However, there were some drawings that could not be classified in any category, since they were too abstract and did not have a specific shape, only color. Figure 2 shows a symbolic representation of elements recognizable by existing literature and correspond to elements of nature, in addition to making a correct spatial representation. Oppositely, other drawings (see Figure 3) do not contain a spatial representation that is attached to reality, even though they do include biotic elements.

**FIGURE 4:**
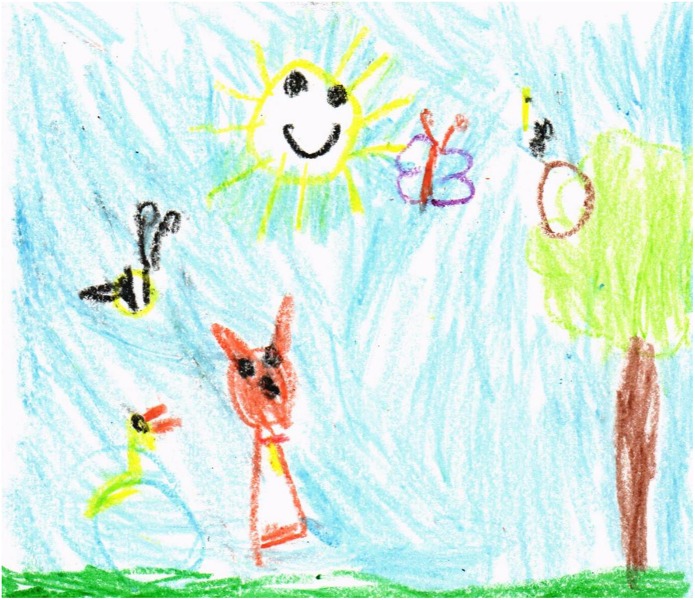
Drawing with support system.

Most of the graphic texts made by the participants show support systems that can be interpreted mostly in an easy way, as they represent objects from the outside world expressed in a realistic way according to their developmental stage, such as those presented in Figure 4. In this way, many of the symbols are understandable without need for interpretation by the participant; also, some texts as a whole are easily recognizable and decipherable by the reader.

The drawings also showed that children drew facial expressions on some natural elements (Figure 4). Whenever this happened, they were always smiling faces, even on animals. It should be noted that the symbols that represent human beings are found in the lower quadrants, which represent the position where terrestrial beings move and live, whereas the superior quadrants are used either for extensions of large terrestrial elements such as trees or mountains, or for the location of birds, celestial bodies, and abiotic elements such as clouds.

Furthermore, in the graphic representations, 13 participants drew people, representing themselves or other relatives as part of nature. However, when questioned if they felt part of nature, 81 participants answered positively, which indicates that participants include an environmental identity as a part of their self-definition, despite most not expressing it in their drawings.

## Discussion

The study was based on a discursive analysis of the drawings produced by preschool children with the purpose of observing the elements that make up their understanding of nature. The use of art as tool of data collection proved to be an effective technique for the externalization of the participants’ ideas regarding the studied phenomenon. This demonstrates children’s deep understanding of the concept of nature.

For the analysis, the components of the productions were identified and granted keywords in order to determine the participant’s perception of the subject. This way, ambiguities when interpreting the products were reduced, which is what Macdonald (2009) describes as a holistic approach of the representations that the participant has regarding the understanding of the phenomenon (Deguara and Nutbrown, 2018). The analysis was also based on the visual discourse analysis proposed by Albers (2007), which states that the support systems present in drawings help interpreters understand the meanings the creator intended to depict by decoding the semantics of the symbols used through a cultural tenet.

The analysis showed that a general idea of what nature represents includes trees, birds, and food, which coincide with the areas where participants live; however, the number of endemic elements of the ecosystem in which they live was relatively low, omitting fauna and flora typical of their region such as snakes, iguanas, biznagas, or pitayas. This corresponds to the research of Bolzán et al. (2014), where it is established that the place of residence is not reflected in the way of drawing nature, but in learning from formal environmental education (2014: 38).

It was observed that productions contained elements of the environment that could be found in the nearby areas and were immediately recognizable by the participants, such as trees, flowers, birds, grass, butterflies, clouds, palm trees, mountains, fruits, snakes, or the sun. Additionally, there was a large number of symbols that represent elements not native of the environment where the participants live, including lions, giraffes, elephants, jungles, and forests, which can be attributed to the contact of the participant with material that contained this type of representations of nature (Bolzán et al., 2014). This supports the statement that knowledge of the phenomenon helps to create productions in greater detail and with a greater number of elements, as shown by the study by Barraza (1998), who affirmed that the perception of children is influenced by knowledge, age, and their ability to draw.

The visual representations themselves enclose properties that cannot be reduced to mere substitutions of objects in real life (Banks, 2007), and representations of nature by preschoolers show categories of natural elements such as animals, plants, food, abiotic factors, locations, and people (Alerby, 2000; Ulker, 2012; Özsoy, 2012).

According to the studies of Zabulis and Orphanoudakis (2001) and Kress and Van Leeuwen (2007), children’s points of interest are indicated by the positioning of objects through the paper and attract the viewer’s attention to these spaces. They explain that this selection of accommodation is itself qualitative information by the creator. This can be seen in the productions made by the participants that make spatial use according to the symbols they represent. This is how the sun and clouds were drawn in upper quadrants or “above,” and trees or plants in the lower quadrants or “below.” However, there were graphic texts that did not take into consideration the social conventions of “above” and “below” within the productions. This corresponds to the statements of Zabulis and Orphanoudakis (2001) who specify that the content of an image includes form, color, or intensity as well as spatial organization. However, some visual elements reside in the perceptual domain.

Additionally, it could be observed that there are, among the drawings, abstract and metaphorical creations about real-world referents, like what represents a tree, a dog, a person, and so on, with some participants. This also coincides with developmental theories that explain that, depending on the level of maturity of the child, these referents will get closer to more accurate representations or to the natural world. Bell’s (2003) visual content analysis explains that objects are mutually exclusive and should not be taken “literally,” but as a manifestation of reality by the author.

None of the representations made reference to contaminated environments, and only a few showed human intervention that was different to the studies by Alerby (2000); Shepardson et al. (2007), Özsoy (2012); Ulker (2012), and Bolzán et al. (2014). The study carried out by Pasca et al. (2019) was also considered, where they sought to determine the way in which people categorized the environments according to the nature they saw in some pictures. One of the findings reported in this study was that people did not categorize as natural environments all photos where human presence or built places were depicted. This coincides with our study, where we found that children’s representations of nature did not include any built environment in their drawings.

As mentioned before, the main objective of the study was to offer empirical evidence obtained through research methods different from the traditional ones in psychology and environmental education, i.e., qualitative approaches. This allowed us to construct knowledge from the child’s perspective using their abilities. Further improvements to this approach are expected to enhance the understanding of children’s representation of nature. One such improvement could be introduced by adding new questions to include in the proposed organization of perception of nature. These questions should consider family habits, outdoor and indoor activities, and time spent watching television or using the internet, if the children have traveled with their family, among other relevant lifestyle aspects. As mentioned by Carrus et al. (2015), the educational spaces and the experiences that children live in their school environments are qualitatively different from the experiences they live in their homes; therefore, environmental perception can be influenced by these other contexts. Regardless, the work presented here is also limited by classical considerations of qualitative studies, in which findings cannot be generalized or extended to wider populations even if they are similar. Thus, a high degree of certainty cannot be assured.

The dominant tradition in environmental psychology research is to use scales (self-reports) developed in studies with adults and “adapt” them to the study of children; thus, we sought to carry out an investigation adapted from the start to the study of the early stages in human development. Our purpose was to research environmental perception and environmental identity in early childhood; in such manner, the results of this work will shed light on the understanding of an important methodological qualitative approach and contribution. Furthermore, results obtained here may have implications for understanding the concept of nature in early childhood, which may be useful in practical decision making for curricular design of environmental education programs and psychoeducational intervention in the future.

## Data Availability Statement

The datasets generated for this study are available on request to the corresponding author.

## Ethics Statement

The studies involving human participants were reviewed and approved by the Comité de Ética en Investigación de la Universidad de Sonora. Written informed consent to participate in this study was provided by the participants’ legal guardian/next of kin.

## Author Contributions

All authors contributed to the conception, design, and interpretation of the studies. BF was responsible for the planning and design of the study. NB was responsible for the collection and analysis of data. RV was responsible for the interpretation of data and writing. All authors contributed to revising it critically for important intellectual content and gave final approval of the final draft.

## Conflict of Interest

The authors declare that the research was conducted in the absence of any commercial or financial relationships that could be construed as a potential conflict of interest.
